# The Effect of Isometric Exercise Position on the Effectiveness of Isolated Work of the Thigh Flexor Muscles Based on the Results of the sEMG Study

**DOI:** 10.3390/clinpract14060174

**Published:** 2024-10-22

**Authors:** Joanna Zyznawska, Grzegorz Frankowski, Ewa Wodka-Natkaniec, Joanna Skoczek

**Affiliations:** Department of Physiotherapy, Institute of Physiotherapy, Faculty of Health Sciences, Jagiellonian University Medical College, 30-688 Krakow, Poland; joanna.zyznawska@uj.edu.pl (J.Z.); grzegorz.frankowski@uj.edu.pl (G.F.); joanna.skoczek@uj.edu.pl (J.S.)

**Keywords:** activity muscles, lower limb, hamstrings, biceps femoris, sEMG, sport, injury

## Abstract

Backgroud: The main function of the hamstring muscles is to bend the knee joint and support the function of the hip extensors. Their frequent injuries are the result of overload related to, among others, dynamic running or jumping, and inadequate preparation for athletics activities. The asymmetry of the work of individual flexor muscle groups is clearly marked in the case of valgus or varus of the knee joint, i.e., in different positions of the lower limb. The aim of the study was to determine the position and form of a rehabilitation exercise in which an isolated group of muscles flexing the knee joint will show the greatest bioelectrical activity. Methods: The study involved 25 students of the Jagiellonian University Medical College. The students were aged 20–26. The average age was 22.9 (±1.4). The study participants included 17 women with an average age of 23.0 (±1.1) and 8 men with an average age of 22.6 (±1.9). Women constituted 68% and men 32% of all respondents. All participants agreed to participate in the study. Surface electromyography measurements in both lower limbs provided an initial number of 50 cases. The activity of the knee flexor muscles during isometric contraction with resistance was measured in correlation with three foot and lower leg settings: internal rotation, neutral position, and external rotation. The bioelectrical activity of the semitendinosus muscle is significantly higher (*p* < 0.01) in the internal rotation position than in the neutral position of the lower leg, while the bioelectrical activity of the biceps femoris muscle is inversely higher (*p* < 0.01) in the external rotation position than in the neutral position. Results: The results are significant for both average and maximum values of muscle stimulation. During isometric contraction with resistance, the semitendinosus muscle shows the greatest bioelectrical activity in the internal rotation position of the lower leg and foot, and the biceps femoris muscle in the external rotation position of the lower leg and foot. Conclusions: The above information has important implicational applications when improving isolated groups of hamstrings. In the future, this may contribute to more effective rehabilitation of patients with injuries of the muscles described in the article.

## 1. Introduction

Weakness of the knee flexor muscles is one of the most common and problematic dysfunctions in the lower limb. This is usually a consequence of overload or injuries to the posterior group of thigh muscles. Injuries to the hamstrings and, particularly, the biceps femoris muscle are among the most commonly reported injuries in sports. They are common in high-speed disciplines such as football, athletics, or sports that require interval sprinting. These injuries are often associated with a significant decrease in strength and muscle mass and, thus, long-term elimination from sports [1,2]. Reports in the literature indicate that injuries of the biceps femoris muscle are characterized by a very high recurrence rate. Re-injury may be caused by the athlete’s premature return to sport, incomplete rehabilitation procedures, or the omission of preventive prophylaxis after the resumption of training [2,3,4]. The main cause of this situation is indicated as muscle strength deficits of the biceps femoris [5]. Also, the most important risk factors include a disturbed balance of moments of force between both limbs and between agonists and antagonists within the same limb [6]. It was confirmed that the degree of stability of the knee joint depends on the hamstring reflex reaction in the event of posterior–anterior tibial translation shifts [7,8,9]. Aagaard et al. [10] suggest that a decrease in the strength of the hamstring muscles in relation to the strength of the quadriceps muscles may be associated with the risk of more frequent knee joint injuries.

Previous studies in the past on the comparison of rehabilitation programs after acute injury of the sciatic and shin muscles showed that the lack of properly conducted physiotherapy in over 50% of cases is the cause of re-injury within the first 2 months after the injury and in 70% after the injury in relation to the group undergoing proper rehabilitation, where this indicator was several percent [11].

Muscle weakness of the posterior thigh muscles may also occur as a result of inactivity, injuries, and pain in the knee joint, or it is associated with varus or valgus of the knee joints [12,13]. Behrens et al. [9] suggest that an exercise causing hamstring muscle fatigue results in a decrease in muscle–nerve function and greater posterior–anterior shifts of the tibia, which indirectly contributes to an increase in the incidence of ACL injuries in women. Authors Johansson et al. and More et al. conclude that the biceps femoris muscles contribute to good knee joint stability and protect the anterior cruciate ligament during the translation of the tibia relative to the femur [14,15].

An important action in conditioning the proper function of the thigh flexor muscles is the implementation of proper prevention, preventive measures, and a properly selected training and physiotherapy program. The key to rebuilding the lost function and strength is to strengthen the knee flexor group in a selective way, paying attention to forcing the activity of a specific group of thigh muscles. A study by Friemert et al. confirms that the activation of the hamstring muscles results in better stability of the knee joint [7]. There are many known exercises that affect the effective activation of the entire group of sciatic and shin muscles [4,16,17], but the research included in this paper indicates the possibility of isolated activation, especially in the initial phase of rehabilitation.

The aim of the study was to investigate the bioelectrical activity of the lateral group of knee flexor muscles (semitendinosus, semimembranosus) and the biceps femoris muscle during isometric contraction in isolated, unilateral work, depending on the position of the lower leg and foot of the lower limb. An equal goal was to determine the optimal position in which these muscles in the isolated version work most effectively.

Research questions were asked:

1. Is there a difference in the bioelectrical activity of the lower leg flexor muscles, depending on the position in which they work?
2. Is it possible to find a position in which the semitendinosus and semimembranosus muscles (lateral femoral group) are more active than the biceps femoris muscle (medial femoral group), or vice versa?
3. Is it possible to effectively exercise only one group of lower leg flexor muscles?

## 2. Materials and Methods

The study involved 25 students of Physiotherapy at the Faculty of Health Sciences of the Jagiellonian University Medical College, aged 20–26, with an average of 22.9 (±1.4). There were 17 women (68%) and 8 men (32%) in the study group. The study was conducted in accordance with the Declaration of Helsinki and approved by the Committee for Scientific Research Ethics of the Jagiellonian University Medical College (118.6120.75.2023, date: 22 September 2023).

Selection of participants

The inclusion criteria included: student status of the Faculty of Health Sciences of the Jagiellonian University Medical College; age over 18 years of age; no contraindications to electromyography; good health; no pain and no previous injuries within the lower limb; provided written informed consent. The exclusion criteria were: recent injuries to the lower limb, pain and painful cramps in the examined muscles during movement, inability to maintain contraction for at least 5 s, and lack of consent to participate in the study. The tests were performed at the Department of Physiotherapy of the Jagiellonian University in Krakow, with the use of measuring equipment of the Laboratory for Testing Vital Functions CDT CARD.

Before taking the measurements, all participants were informed about the course of the study and filled out an anonymous questionnaire in which information on physical activity and past injuries was collected. Each participant signed an informed and voluntary consent to participate in the research. All of them described their physical fitness as quite high. Due to past injuries, one case for the biceps femoris muscle and two cases of pain in the semitendinosus muscles were excluded. Ultimately, this provided a number of 48 cases of semitendinosus muscle and 49 cases of biceps femoris muscle.

Sequence of tests

Twenty-five participants were recruited. The study group consisted of students of the Jagiellonian University Collegium Medicum, majoring in physiotherapy. The main inclusion factor was the lack of contraindications to physical activity and the absence of injuries in the examined muscles. Before starting the test, each participant had a procedure to check the maximum resistance of the subject. Participants started the trial with a random lower limb. Each participant was asked to lie on their front, hands clasped under the forehead, knee joint bent to approximately 90 degrees, and foot and lower leg in internal rotation. After completing the test, the subject assumed another position: lying on the front, hands clasped under the forehead, knee joint bent to approximately 90 degrees, and foot and lower leg in a neutral position, without rotation. The next test involved lying on the front, hands clasped under the forehead, knee joint bent to approximately 90 degrees, and foot and lower leg in external rotation. First, all three variants of the test were performed on one leg, then on the other. The breaks between each trial were 1 min.

Test conditions

The tests were always performed in the same room (dimensions: 20 m long; 10 m wide; 4 m high) and air-conditioned with a constant temperature of 22 °C. In order to eliminate potential risk factors, all nearby objects that could disturb the subject and interfere with the sEMG signal were removed. There was always a researcher nearby to provide security.

Measurement procedure

Each participant was measured by the bioelectromyographic activity of the semitendinosus and biceps femoris muscles in both limbs. The study focused on checking in which position of the lower leg these muscles work most effectively during isometric contraction with resistance. Each of the studied muscle groups was treated as a separate case. All subjects were placed in the same starting position, lying forward. In addition, the BIODEX System was used to provide participants with equal conditions for isometric muscle contraction.

Surface Electromyography (sEMG)

Before the start, each participant was informed about the course of the study. The sEMG procedure was conducted in accordance with standard recommendations for this type of examination [18]. The study used superficial electrodes, pregelatinized with silver chloride recommended by SENIAM. The electrodes were disposable with a diameter (conduction area) of 1 cm [19]. In places where the electrodes were applied, the skin was cleaned with salicylic spirit and left to dry. Electrodes were placed along the muscle fibers of the muscle bellies.

Then, Noraxon ULTIUM sensors were connected to the electrodes and a procedure was conducted to check the correctness of the recording/signal.

The acquired signal was processed using dedicated Noraxon MyoResearch software (MR3: 0043-95e0, Version 3.18.46). RMS normalization and ECG signal reduction were used to process the signal. During signal collection, a recording window of 110 ms was defined. This indicated the absence of artifacts, and that the baseline noise did not exceed 5 μV within 5 s, which is a correct result for each subject.

A Noraxon ULTIUM device was used to perform the superficial electromyography (sEMG) procedure. It was used to measure and record the stimulation of the semitendinosus muscle (as a representative of the muscles’ flexors of the lateral femoral group) and the biceps femoris muscle. All results were recorded. From the results obtained, reports were created which included: test duration [s], mean excitation value [μV], and maximum excitation value [μV]. Then, the average and maximum values were compared depending on the position in which the muscle worked.

What is important is that, due to the specificity of surface electromyography, the signal received came directly from the semitendinosus muscle, which lies superficially in relation to the semimembranosus muscle. These muscles are agonists; they perform the same function [20]. The results presented below describe only the semitendinosus muscle.

Starting positions

Each participant was tested in three starting positions to see how the rotation of the lower leg and foot affected the activity of individual muscles as seen in the sEMG recording.

Position No. 1: Lying forward, hands clasped under the forehead, knee joint bent to about 90 degrees, foot and lower leg in internal rotation.

Position No. 2: Lying forward, hands clasped under the forehead, knee joint bent to about 90 degrees, foot and lower leg in a neutral position, without rotation.

Position No. 3: Lying forward, hands clasped under the forehead, knee joint bent to about 90 degrees, foot and lower leg in external rotation.

System BIODEX

In all positions, the subject actively performed isometric contraction against the resistance that was applied to the distal part of the lower leg with the BIODEX device at the level of 50–70% of the maximum resistance, calculated earlier with the use of the Biodex System. Each participant made three attempts in each of the starting positions. Isometric contraction was performed with the same, constant resistance, identical for each trial.

The person monitoring muscle activity in a computer program started recording data 2–3 s after the subject initiated contraction. The subject was asked to hold in a constant isometric contraction for 5 to 10 s. At the same time, he could not lift his pelvis and other parts of his body, except one of his lower legs from the ground. On the STOP command, the subject relaxed his muscles, and the person operating the computer program finished recording. There was a 1 min break between the stages. Reports were made from each trial containing a 5 s record of muscle stimulation.

For a particular muscle, in each of the initial positions (internal rotation, neutral position, and external rotation), collective reports were prepared, while the average [μV] of the muscle work value from three trials and the maximum [μV] muscle work value from three trials were calculated.

The collection of these data allowed them to be compared, and the statistical significance of the parameters was studied.

Develop the results

In order to develop the results, the following parameters were used: mean results from three trials for the mean value of muscle excitation and mean results from three trials for the maximum value of muscle excitation for all tested initial positions: internal rotation, external rotation, and neutral position of the lower leg and foot position of the examined lower limb. It was checked whether the results obtained in rotational positions differed significantly from those obtained in the neutral position.

Statistical analysis

The evaluation of the results was made using the Statistica 13 program from StatSoft. Compliance with the normal distribution was tested using the Shapiro–Wilk test. To determine the statistical significance of the studied traits, the parametric test of the Student’s *t*-test for dependent samples was used. The Student’s *t*-test was performed for mean values in the position of both rotations and the neutral position for both muscle groups.

The level of statistical significance was α = 0.01 for all tests performed. Results at the level of *p* < 0.01 were considered statistically significant.

## 3. Results

### 3.1. Mean Values of Semitendinosus Muscle Excitation (Figure 1)

In the study group, in Position 1 (internal rotation), the mean was M = 240.5 μV (±105.5; min = 91.5; max = 617.7); in Position 2 (neutral), the mean was 211.5 μV (±82.0; min = 70.0; max = 425), for 48.

The result of the Student’s t-parametric test for the dependent samples indicated the statistical significance of the differences for the analyzed mean values (*p* = 0.000031), i.e., (*p* < α) for the higher mean muscle excitation in internal rotation in relation to the neutral position.

### 3.2. Average Activity of the Biceps Femoris Muscle (Figure 2)

In the study group at the time of Measurement 1, the average activity of the tested muscle was 182.1 ± 70.5 μV, the highest recorded value was 363.3 μV, and the lowest was 55.3 μV. In contrast, during Measurement 2, the average activity of the muscle tested was 207.7 ± 79.9 μV, the highest recorded value was 431.3 μV, and the lowest was 62.1 μV, for n = 49.

The result of the Student’s t-parametric test for the dependent samples indicated the statistical significance of the differences for the analyzed mean values (*p* = 0.000003).

### 3.3. The Maximum Values of Excitation of the Semitendinosus Muscle (Figure 3)

Then, the Student’s *t*-test was performed for maximum values in all positions. The following results were obtained for the semitendinosus muscle: in the internal rotation position M = 366.1 [μV] (SD = 170.0: min = 142; max = 1004),) while in the neutral position, it is equal to M = 325.3 [μV] (SD = 133.2; min = 103.9; max = 767.3), for n = 48.

### 3.4. Maximum Activity of the Biceps Femoris Muscle (Figure 4)

In Measure 1, the average peak activity value of the muscle tested was 277.7 ± 107.7 μV, the highest recorded value was 551.3 μV, and the lowest was 83.5 μV. In Measurement 2, the mean peak activity value of the muscle tested was 311.4 ± 116.7 μV, the highest recorded value was 630.7 μV, and the lowest was 87.9 μV, for n = 49.

Similarly to the average values, the maximum values of muscle stimulation also differ significantly from each other, in favor of rotational positions. Statistical significance was obtained in all cases: semitendinosus muscle *p* = 0.000053 (*p* < α) for internal rotation, biceps muscle: *p* = 0.000006 (*p* < α) for external rotation.

## 4. Discussion

Testing the bioelectrical activity of muscles using sEMG, due to its non-invasiveness [21,22], has recently become a very useful research and diagnostic element in the work of a physiotherapist. Often, during the rehabilitation process, the question arises in which position to strengthen a specific muscle selectively while not unnecessarily activating other muscle groups co-responsible for the same function. This is especially a problem when a group of muscles cooperating during the performance of a common function dominates the weakened muscle and makes it difficult to strengthen it, taking over the entire role. In scientific studies, many attempts have been made to indicate effective exercises to strengthen the thigh flexor group, but the main focus has been on their type and joint strengthening, rather than selection for the lateral and medial groups. [17,23,24] Research suggests that posterior thigh muscle activity is greatest during squat lift (concentric phase), which is strongly associated with weightlifting, and reaches maximum activity in the range of 50–70 degrees of knee flexion [23]. Blanpied reports that a significant increase in the activity of the sciatica and shin muscles is observed when performing squats on the semi-free Hack Squat leg muscle exercise. It has also been noted that deep and semi-full squats are equally effective in activating the sciatic-shin muscles [24]. Marušič and Šarabon point out the beneficial use of Nordic exercises (controlled descent of the upright trunk from a straight kneeling position) for sciatica and shin muscles in the prevention and rehabilitation of injuries to this muscle group. Researchers have proven that eccentric Nordic fall exercises provide the highest peak activity of the knee flexors, thus effectively increasing their strength and the length of the fascia of the biceps femoris muscle [17].

Although, in the literature, you can find examples of the effective strengthening of the entire group of sciatic-shin muscles, there is little information on how to activate the biceps femoris or semitendinosus muscle in an isolated way. This is especially useful in cases where there is a muscle imbalance within the ischioshin group. Selective activation of the muscles of a specific side is conducive to the effective elimination of the imbalance of the thigh flexor group and restoration of symmetry of the work of the semimembranous and semimembranous muscle with the biceps femoris muscle. This can be particularly useful in conducting rehabilitation after weakness of one of the muscles; for example, in posture defects, such as varus or valgus knees in children, as well as among athletes who want to increase the strength and mass of a specific muscle from the ischioshin group after an injury in an isolated way.

The professional literature describes studies that evaluated the effect of joint positioning on the strength of the muscles of the lower limbs and proves that changes in the position of the ankle, knee, or hip joint can affect the change in muscle activity during movement. These studies, conducted on the basis of the analysis of the quadriceps femoris muscle, suggest that it is possible to isolate the work of individual muscles from the entire group of agonists [25,26,27]. Signorile et al. showed that the modification of the position of the foot or knee joint during movement causes selective activation of the quadriceps muscle and affects the change of activity within it [28]. Stoutenberg et al. proved that internal rotation of the foot provides the greatest activation of the vastus medialis and vastus lateral muscles, while external rotation causes the greatest activation within the rectus femoris muscle [27].

In a study on the muscles of the posterior thighs, the authors Zebis et al., using sEMG, showed that different strengthening exercises can have different effects on individual muscles of the ischioshin group [29]. It was proven that the semitendinosus muscle significantly dominated the biceps femoris muscle in the following exercises: “Romanian Deadlift”, consisting of raising and lowering the stay, with a properly selected weight, and “Kettlebell Swing”, consisting in swinging the kettlebell weight forward from the level of the knees to the level of the shoulders. During these exercises, the knee and hip joints flexed and straightened while keeping the back straight. The findings of this study are valuable for an advanced group of athletes, but the exercises presented will be primarily for athletes rather than convalescents of injury in the early stages.

In the publication entitled: Kinematic and Electromyographic Analysis of the Ask-ling LProtocol for Hamstring Training [30], the authors discuss the topic of training the hamstring muscles with the help of the L Protocol proposed by Askling et al. [31]. The effect of various exercises on individual muscles, such as biceps femoris, semitendinosus, gluteus maximus, and quadriceps femoris, was checked using sEMG. This protocol includes three exercises that activate eccentric contraction in the hamstrings in different ways. The first of them, The Extender, is performed to make the sciatic and shin muscles more flexible. The second, The Diver, aims to strengthen the sciatic-shin muscles and stabilize the torso. The third exercise, The Glider, during which an eccentric contraction is performed, also serves to strengthen the muscles. Studies have shown that only two out of three exercises (The Diver and The Glider) led to a marked activation of the hamstrings. The authors point out that a particular exercise protocol has a great advantage in post-traumatic rehabilitation of the sciatic-shin muscles compared to traditional strengthening exercises due to the fact that muscle strength is built in a different way, e.g., through eccentric contraction. This is certainly a valuable indication as to advanced rehabilitation exercises, but next to them, it is worth considering exercises with traditional reinforcement as shown in this work, if only because of the ease of their performance and low load applicability in the initial phase of post-traumatic rehabilitation. The cited publication also shows that the hamstrings are strongly activated during isometric contraction proposed in the discussed research.

In another study by Bourne et al. [32], strengthening exercises enabling selective activation of the sciatic-shin muscles are presented. The study procedure involved measuring the activity of the biceps femoris and semitendinosus muscles using sEMG and was conducted during the concentric and eccentric phases of 10 different strengthening exercises. One of them was the Nordic Curl exercise, which consisted of kneeling with both legs, with stabilization of the lower limbs in the area of the ankle joints, set in a neutral position, and slow leaning forward. This exercise causes eccentric contraction of the hamstrings and mostly activates the medial muscles of the posterior group of thighs.

A review of the medical literature on exercises to strengthen the posterior thigh muscle group by Bourne et al. summarized the considerations of various authors, saying that the activation of individual muscles of the ischioshin group depends on different exercises [16]. Some authors present that the semitendinosus and semimembranosus muscles are best activated during the Nordic Curl exercise, while others show that the best exercise may be the Leg Curl exercise (an exercise lying forward on a weighted machine) in the eccentric phase [33].

Limits of the study

Our study focused on a specific young age group and an academic population that is physically active but does not exercise professionally. On the one hand, the advantage of the conducted research is the fact that it concerned a homogeneous age group and the lack of a diverse population, which may provide a clean result for these people. On the other hand, this may constitute a limitation when the results are implied for other, different age groups or populations. Therefore, the results should not be generalized for every age group or population. In further future research, it is necessary to consider expanding the research to include different age categories (e.g., older people) and different populations (professional athletes, physically inactive ones). Another limitation may be the small group of respondents (25 participants) who ultimately decided to participate in the study. We recommend increasing the number of subjects in future work. It should be remembered that the results obtained are intended for physiotherapists, sports medicine doctors, and trainers dealing with physically active people and amateur sports. It should be borne in mind that the results of our research are aimed at people in the early phase of recovery after damage in the hamstring area. This is important because it suggests how to optimally start working in early therapy, seemingly the shortest, but constituting the starting point for further stages of improvement.

Summing up the considerations of various authors, it can be noted that the sciatic shin muscles were best activated in exercises with eccentric contraction. However, none of the studies compared the bioelectrical activity of the muscles that occurs during the simple isometric contraction proposed in the present study. There is also no example of easy and quick muscle activation divided into lateral or medial groups of the thigh flexor muscles.

This study is one of the first to assess the bioelectromyographic activity of the knee flexor muscles depending on the external or internal position of rotation of the lower leg relative to the thigh, which may be of significant importance for people after hamstring injuries and suffering from intermuscular imbalance, among others, in relation to transplants for ACL repair.

More physiotherapists, coaches, and sports scientists dealing with early-stage injuries of the posterior thigh muscles should include lower limb exercises in their training protocols, including rotation of the lower leg (external or internal), depending on the weakening of the activation of the lateral or medial muscles of the posterior thigh group. Indeed, this aims to speed up recovery and reduce the risk of secondary injury in this area thanks to targeted training activities.

## 5. Conclusions

During isometric contraction with resistance in knee flexion at an angle of about 90 degrees, there is a significant difference in the bioelectrical activity of the lower leg flexor muscles depending on the position of the foot and lower leg.

The greatest bioelectrical activity in the isometric contraction of the semitendinosus and semimembranosus muscle in the flexion position of the knee joint occurs in correlation with internal rotation of the lower leg and foot.

The greatest bioelectrical activity in the isometric contraction of the biceps femoris muscle in the flexion position of the knee joint occurs during external rotation of the lower leg and foot.

A better understanding of the relationship between the position of the lower limb and the activity of the hamstrings muscles makes it easier to predict optimal patterns of rehabilitation exercises in the case of disorders of these muscles, which is important implications during physiotherapy and training.

## Figures and Tables

**Figure 1:**
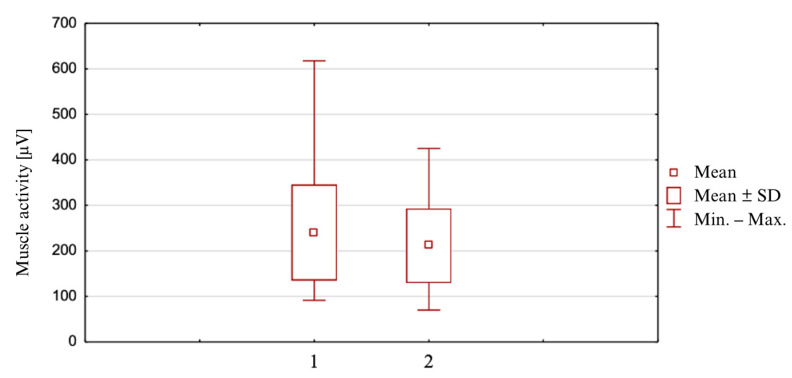
Mean values of semitendinosus muscle excitation in two starting positions. 1—internal rotation position, 2—neutral position.

**Figure 2:**
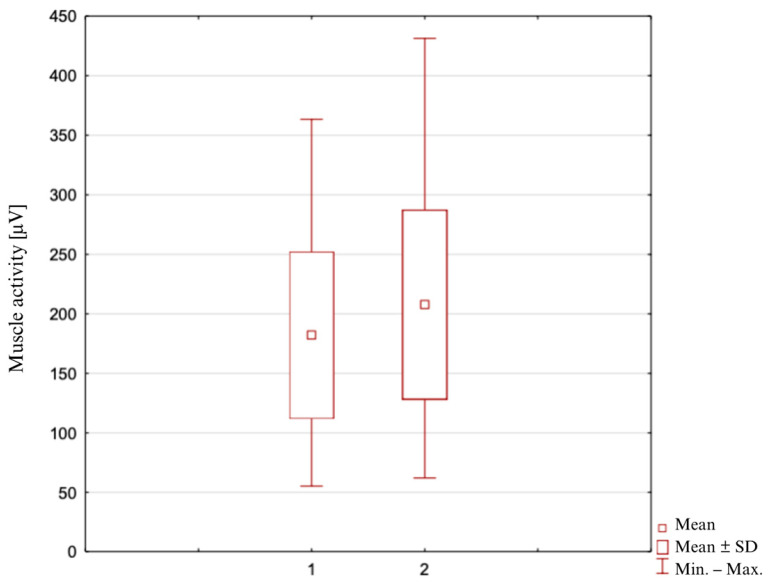
Average activity of the biceps femoris muscle during the measurement: 1—in the neutral position of the lower leg; 2—in the position of maximum external rotation of the lower leg.

**Figure 3:**
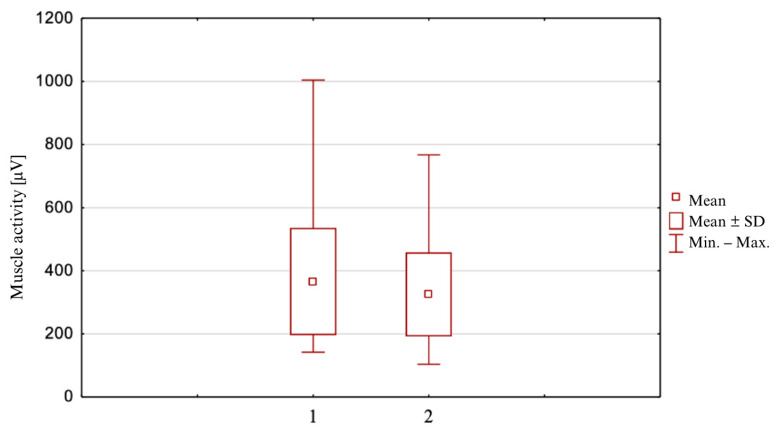
The maximum values of excitation of the semitendinosus muscle in two starting positions. 1—internal rotation position; 2—neutral position.

**Figure 4:**
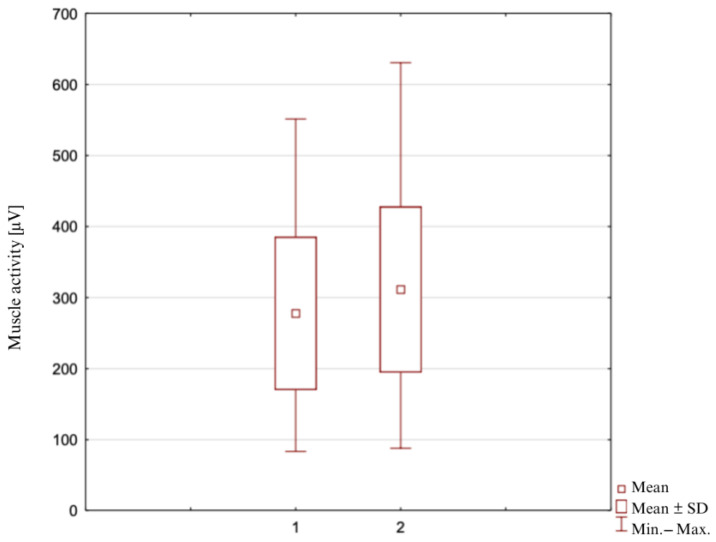
Maximum activity of the biceps femoris muscle during measurement: 1—in the neutral position of the lower leg; 2—in the position of maximum external rotation of the lower leg.

## Data Availability

The data supporting this study’s findings are available from the corresponding author upon reasonable request. The data are not publicly available due to privacy and ethical restrictions.
